# Dielectric Engineering to Suppress Cell-to-Cell Programming Voltage Interference in 3D NAND Flash Memory

**DOI:** 10.3390/mi12111297

**Published:** 2021-10-22

**Authors:** Woo-Jin Jung, Jun-Young Park

**Affiliations:** School of Electronics Engineering, Chungbuk National University, Chungdae-ro 1, Chungbuk, Cheongju 28644, Korea; jwjin@chungbuk.ac.kr

**Keywords:** dielectrics, flash memory, interference, cell-programming, vacuum dielectric, V-NAND

## Abstract

In contrast to conventional 2-dimensional (2D) NAND flash memory, in 3D NAND flash memory, cell-to-cell interference stemming from parasitic capacitance between the word-lines (WLs) is difficult to control because the number of WLs, achieved for better packing density, have been dramatically increased under limited height of NAND string. In this context, finding a novel approach based on dielectric engineering seems timely and applicable. This paper covers the voltage interference characteristics in 3D NAND with respect to dielectrics, then proposes an alternative cell structure to suppress such interference.

## 1. Introduction

NAND flash memory data storage has been developed for decades based on CMOS (complementary metal-oxide-semiconductor) technology [1]. NAND flash consists of memory cells and peripheral circuits. Binary data are ‘programmed’ in memory cells and the data state is ‘read’ with the aid of a logic controller that includes peripheral circuits. The data stored in memory cells can be sustained for approximately 10 years without power supply. Hence, NAND flash is categorized as non-volatile memory (NVM). Device structure of a memory cell is based on NMOS transistors, which inherently contain charge trap layers (CTL) such as Si_3_N_4_ in the gate dielectric. When positive bias (typically higher than 10 V) is applied to a gate electrode of a cell transistor to trigger ‘programming’, electrons are moved from the channel to the CTL by FN tunneling mechanism. In this context, the number of electrons stored in the CTL fundamentally represents the size of the binary data.

Device structure of the cell transistor has aggressively evolved for lower bit-cost. To elaborate, the bit-cost can be reduced by increasing cell packing density and so the size of the cell transistor has been scaled down over a long time. However, with scaling down of semiconductor devices, short-channel effects (SCEs) become worse. Therefore, the structure of the cell transistor has evolved from 2-dimensional (2D) planar FET to 3-dimensional (3D) gate-all-around (GAA) FET to suppress SCEs [2].

However, even though SCEs have been quite effectively controlled by the above-mentioned evolution, other concerns have been raised, such as cell-to-cell interference [3,4,5], which is unwanted potential distribution during NAND operation. When high gate bias (V_G_) is applied to gate electrode so-called word-line (WL), electrons are programmed not only in targeted cell transistors, but also in nearby cell transistors, which are not intended to be programmed.

The interference stems from isolation layers such as inter-layer dielectrics (ILD), which are located between each WL. As cell packing density increases under limited NAND string height and dry etching technology, the ILD becomes very thin. To avoid cell-to-cell interference without noticeable advances in fabrication processing, alternative techniques such as incremental step pulse programming (ISPP), multi-level cells (MLC), triple-level cells (TLC) and even quad-level cells (QLC) have been widely applied to mass production [6,7]. However, NAND flash beyond QLC is difficult to develop because of insufficient threshold voltage (V_T_) distribution. Hence, further advances via conventional techniques will be limited. In this context, guidelines for developing cell transistors to improve cell-to-cell interference should be proposed for better cell packing density of NAND flash devices, but such breakthrough research has been modest so far.

In this paper, dielectric engineering is newly proposed to suppress cell-to-cell interference in a 3D NAND flash. First, there is a discussion about unwanted potential distribution among WLs with respect to thickness, diameter and dielectric constant of dielectrics. Thereafter, a novel cell structure is proposed. Considering that demand for NAND flash has been dramatically increased during the COVID-19 pandemic, this research proposal seems very timely [8].

## 2. Materials and Methods

**Figure 1:**
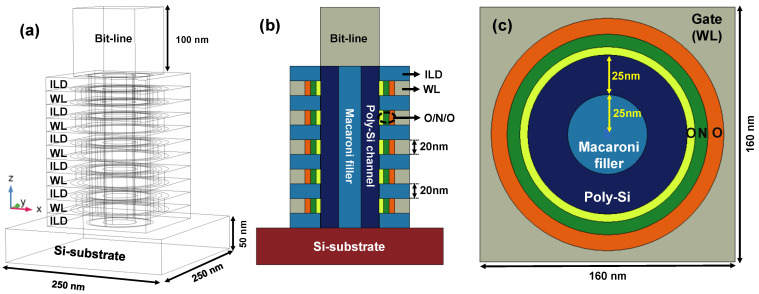
(**a**) Schematic geometry used for 3D simulations. (**b**) Cross-sectional view of structure cut along bit-line (BL) direction (xz-plane in Figure 1a) and (**c**) WL direction (xy-plane in Figure 1a). The O/N/O indicates a gate dielectric composed of SiO_2_, Si_3_N_4_ and SiO_2_, respectively.

The 3D simulator COMSOL was utilized with an AC/DC module to investigate potential distribution in 3D NAND flash, as shown in Figure 1 [9]. Back-bone structure for the simulation was a terabit cell array transistor (TCAT) [10]. Five layers of ILDs, as well as WLs, were stacked on a silicon substrate. In addition, SiO_2_/Si_3_N_4_/Al_2_O_3_ layers were included in a gate dielectric. A poly-Si channel 25 nm thick surrounded the macaroni filler, which was located at the middle of the hole, as shown in Figure 1c. Detailed information on the geometry and materials for simulations is provided in Table 1.

## 3. Results

**Figure 2:**
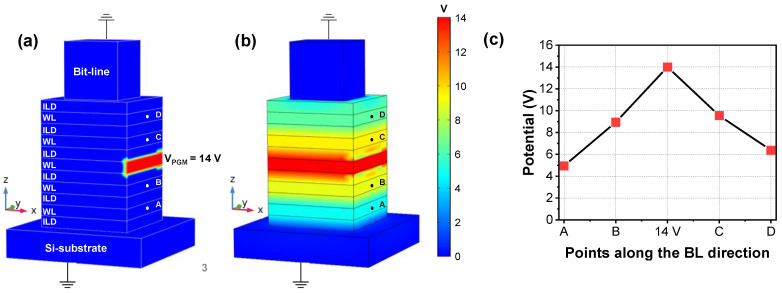
Simulated voltage distribution at (**a**) t = 0 s and (**b**) after saturation. (**c**) Extracted potentials at surface of WLs (points A to D).

To investigate the cell-to-cell interference during the ‘programming’ operation, 14 V DC bias (V_PGM_) was applied to one WL located in the middle of the NAND string, as shown in Figure 2a. During the simulation, both BL and Si-substrate were grounded. After saturation time of approximately 4 fs, the voltage applied to the WL was effectively transferred along the string, as shown in Figure 2b. Even though there are 20 nm ILD layers composed of SiO_2_ among the WLs, extracted voltage drop was approximately 5 V per each WL, as shown in Figure 2c. This result indicates that cell-to-cell interference obviously exists during the cell ‘programming’ configuration in 3D NAND flash.

**Figure 3:**
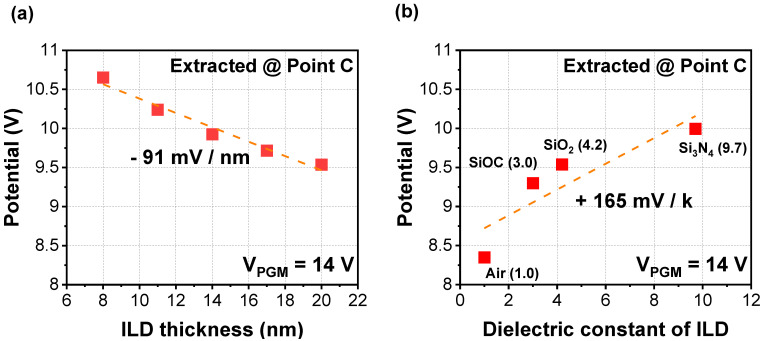
Extracted parasitic potential at nearby WL with various (**a**) thicknesses and (**b**) dielectric constants of ILD when V_PGM_ of 14 V is applied to 3rd WL.

Figure 3a shows cell-to-cell interference characteristic with various ILD thicknesses when V_PGM_ of 14 V is applied to the 3rd WL only. As the ILD thickness decreases, cell-to-cell interference increases with the slope of 91 mV/nm because of increased parasitic capacitance between the WLs. Considering that the ILD thickness becomes thinner as the number of stacks in the 3D NAND flash increases, it is obviously expected that the interference will become severe. Figure 3b shows simulation results with various dielectric materials used as alternatives to the ILD. As the dielectric constant of the ILD decreases, the cell-to-cell interference can be improved because of reduced parasitic capacitance. Hence, applying a low-*k* dielectric such as SiOC as the ILD would be preferred to improve the interference, rather than applying a conventional material such as SiO_2_ [11].

**Figure 4:**
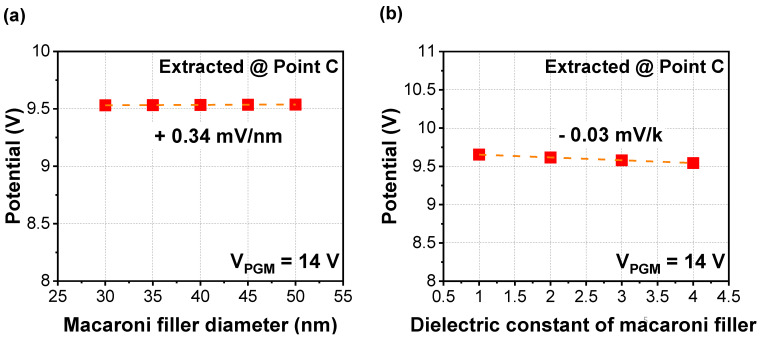
Extracted parasitic potential with various (**a**) diameters and (**b**) dielectric constants of macaroni filler (SiO_2_) when V_PGM_ of 14 V is applied to 3rd WL.

Figure 4 shows the simulation results of cell-to-cell interference for the macaroni filler. As the number of NAND layers increases, variability of the diameter of the macaroni filler in the NAND string also increases. Hence, it is noteworthy to observe the impact of the macaroni filler, as shown in Figure 4a. As the diameter of the macaroni filler decreases, the parasitic potential applied to nearby cells slightly decreased with the sensitivity of 0.34 mV/nm. Hence, a narrower diameter macaroni filler is preferred to minimize cell-to-cell interference. Considering the macaroni filler at low layers of NAND strings shows narrower diameter than filler at high layers, cell-to-cell interference at high layers might be more severe than in cells at low layers. However, there was no remarkable tendency in terms of dielectric constants (Figure 4b).

**Figure 5:**
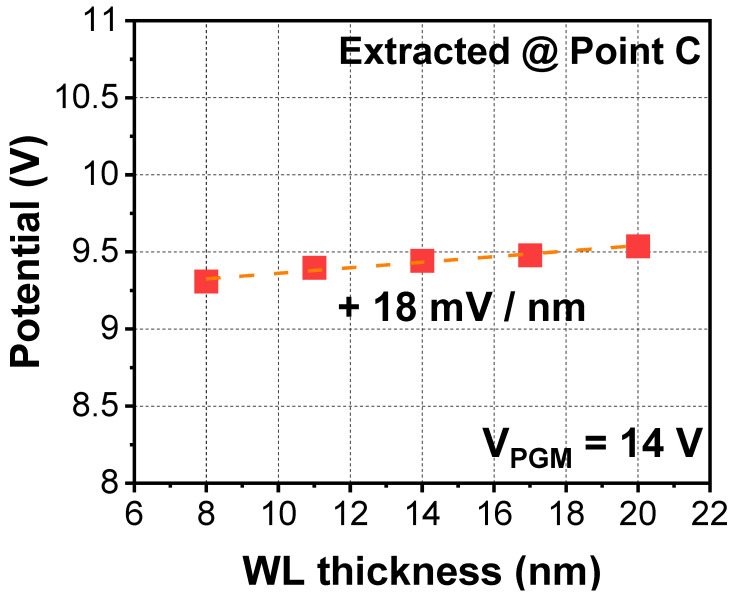
Extracted parasitic potential with various WL metal thicknesses.

Figure 5 shows simulation results with respect to WL (gate) thickness. As the number of stacks in the 3D NAND increases, cell-to-cell interference stemming from the ILD becomes severe, as mentioned above. However, contrary to the case of the ILD, this interference improved as the thickness of the WL decreased. When the WL thickness decreases (i.e., because of gate length scaling), the fringing field effect from the WL decreases and, hence, cell-to-cell interference can be improved [12]. In other words, considering that the WL thickness is being scaled down for higher packing density, additional gate engineering with respect to the interference is not required.

Table 2 provides a summary of the cell-to-cell interferences and sensitivities for the different geometries and materials of dielectrics. It can be seen that the most significant dielectric to be modified to minimize the cell-to-cell interference is the ILD.

**Figure 6:**
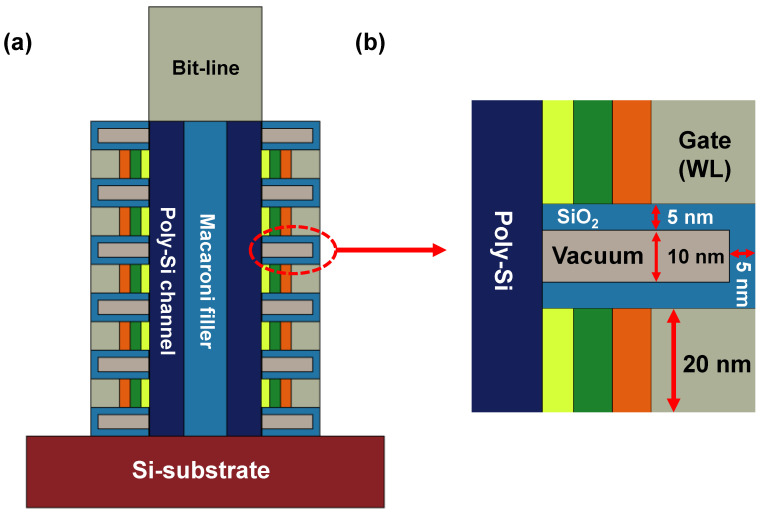
(**a**) Schematic of proposed 3D NAND flash string with vacuum dielectric. (**b**) Magnified image of cells.

In this context, we newly propose a 3D NAND structure to minimize the cell-to-cell interference, as shown in Figure 6. A vacuum cavity was defined inside the ILD and conventional inorganic dielectric material such as SiO_2_ surrounded the cavity [13,14,15,16,17].

**Figure 7:**
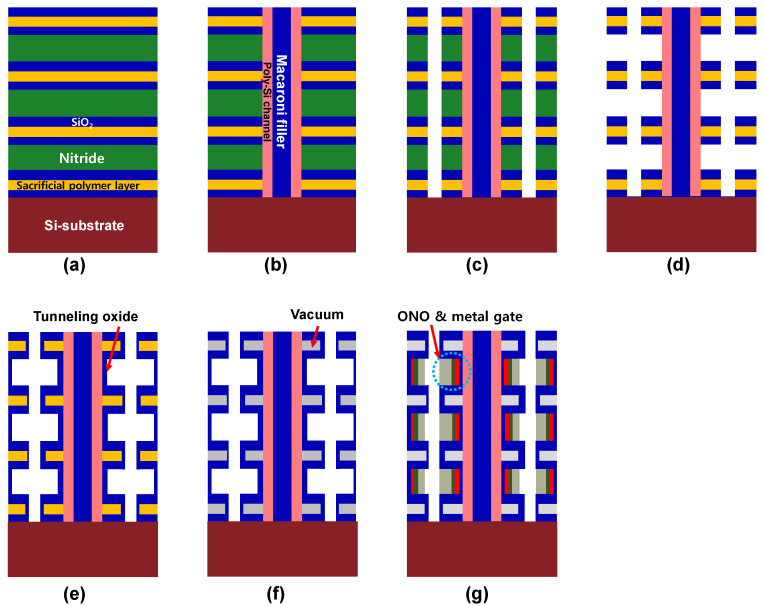
Fabrication process flow of proposed 3D NAND structure containing vacuum dielectric. (**a**) Iterative deposition of oxide, sacrificial polymer, oxide, and silicon nitride layer. (**b**) Dry etching of hole, deposition of poly-silicon channel, and fills the hole by oxide. (**c**,**d**) Dry etching and selective etching of silicon nitride layer. (**e**,**f**) After deposition of tunneling oxide, the polymer layer is thermally decomposed. (**g**) Deposition of charge trap layer, blocking oxide, metal gate, and finally fills inter layer dielectric.

Figure 7 shows the fabrication process flow of the proposed 3D NAND flash. The multi layers composed of SiO_2_/sacrificial polymer layer/SiO_2_ and Si_3_N_4_, were deposited iteratively on an Si-substrate, as shown in Figure 7a. It should be noted that such sacrificial layers can decompose during heat treatment [18,19]. After dry etching, poly-Si was deposited on the sidewall; thereafter, SiO_2_ was filled in as macaroni filler (Figure 7b). Etching of the top side and heavy doping of poly-Si deposition were performed for drain region definition (not shown). Then, dry etching, sacrificial Si_3_N_4_ removal and tunneling oxide deposition were sequentially performed (Figure 7c–e). Thermal annealing to remove the sacrificial polymer layer was performed to form a vacuum dielectric inside the ILD (Figure 7f). Then, Si_3_N_4_ CTL, Al_2_O_3_ blocking oxide and metal gate were deposited (Figure 7g). Finally, the ILD was filled between the nodes (not shown).

**Figure 8:**
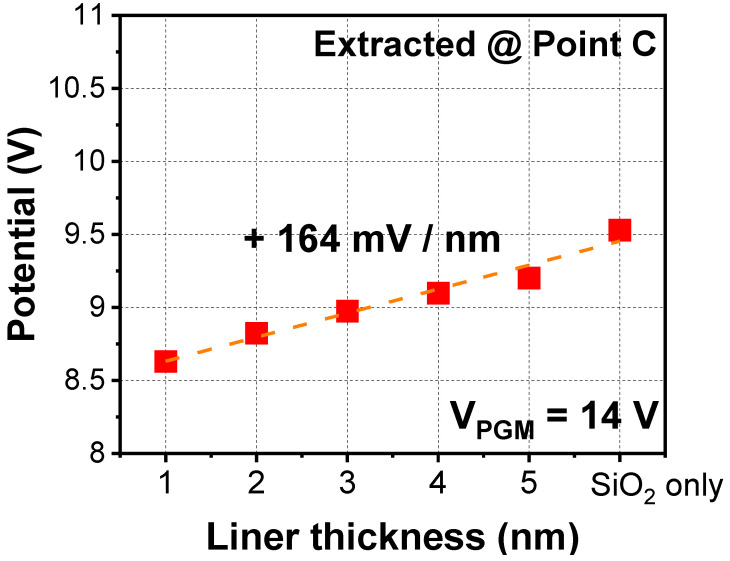
Simulated parasitic potential with various thicknesses of liner surrounding the vacuum cavity.

Figure 8 shows the extracted parasitic potential with various thicknesses of liner composed of SiO_2_. Dielectric constant of vacuum during the simulation was assumed to be 1.0. As the thickness of the liner decreased, the volume of the vacuum increased. The cell-to-cell interference can be improved owing to the lowered parasitic capacitance.

## 4. Conclusions

Simulation studies have been performed to suppress cell-to-cell interference in 3D NAND flash memory during program operation. Voltage interference among the cells was discussed with respect to dielectrics such as inter-layer dielectric (ILD) and macaroni filler. Then, several sensitivities that impact the cell-to-cell interference were extracted and compared. It was found that the most significant dielectric to determine the interference was the ILD. As a result, a novel 3D NAND structure containing a vacuum dielectric inside of the ILD was newly proposed. The cell-to-cell program interference was reduced by aid of the proposed device structure.

## Figures and Tables

**Table 1:** Dimensions and material parameters for 3D simulations.

Geometry	Material	Thickness [nm]	Dielectric Constant	Electrical Conductivity [S/m]
Word-line (WL)	W	20	1	2 × 10^6^
Blocking oxide	Al₂O₃	10	5.7	10^−11^
Charge trap layer	Si_3_N_4_	8	9.7	10^−11^
Tunneling oxide	SiO_2_	5	4.2	10^−11^
Poly-Si channel	Poly-Si	25	4.5	3 × 10^3^
Si-substrate	Si	50	11.7	10^4^
Macaroni filler	SiO_2_	50	4.2	10^−11^
Bit-line (BL) contact	W	100	1	2 × 10^6^
Inter-layer dielectric (ILD)	SiO_2_	20	4.2	10^−11^

**Table 2:** Summary of cell-to-cell interference in terms of dielectrics in 3D NAND flash.

Geometry	Material	Thickness	Dielectric Constant
Inter-layer dielectric (ILD)	SiO_2_	−91 mV/nm	+165 mV/k
Macaroni filler	SiO_2_	+0.34 mV/nm	−0.03 mV/k
Word-line (WL)	W	+18 mV/nm	-
